# The anticancer effect of metformin targets VDAC1 via ER-mitochondria interactions-mediated autophagy in HCC

**DOI:** 10.1038/s12276-024-01357-1

**Published:** 2024-12-03

**Authors:** Minjeong Ko, Jiho Kim, Raudah Lazim, Ju Yeon Lee, Jin Young Kim, Vijayakumar Gosu, Yoonji Lee, Sun Choi, Ho Jeong Kwon

**Affiliations:** 1 Chemical Genomics Leader Research Laboratory, Department of Biotechnology, College of Life Science and Biotechnology, Yonsei University, Seoul, Republic of Korea; 2 Global AI Drug Discovery Center, College of Pharmacy and Graduate School of Pharmaceutical Sciences, Ewha Womans University, Seoul, Republic of Korea; 3 Research Center of Bioconvergence Analysis, Korea Basic Science Institute, Ochang, Republic of Korea; 4 Critical Diseases Diagnostics Convergence Research Center, Korea Research Institute of Bioscience and Biotechnology, Daejeon, Republic of Korea

**Keywords:** Liver cancer, Drug development

## Abstract

Metformin (MetF) is used worldwide as a first-line therapy for type 2 diabetes. Recently, interest in the pleiotropic effects of MetF, such as its anticancer and antiaging properties, has increased. However, the molecular target of MetF and the detailed mechanism underlying its ability to inhibit cell growth through autophagy induction remain incompletely understood. In this study, using an innovative label-free drug affinity responsive target stability (DARTS)-LC-MS/MS method, we discovered that mitochondrial voltage-dependent anion channel 1 (VDAC1) is a novel binding protein involved in the induction of autophagy-related cell death by high-dose MetF in hepatocellular carcinoma (HCC). Computational alanine scanning mutagenesis revealed that MetF and VDAC1 (D9, E203) interact electrostatically. MetF disrupts the IP_3_R-GRP75-VDAC1 complex, which plays a key role in stabilizing mitochondria-associated ER membranes (MAMs), by binding to VDAC1. This disruption leads to increased cytosolic calcium levels, thereby contributing to autophagy induction. MetF also decreased the AMP/ATP ratio and activated the AMPK pathway. Cells with genetic knockdown of VDAC1 mimicked the activity of MetF. In conclusion, this study provides new insights into the involvement of MetF in ionic interactions with VDAC1, contributing to its anticancer effects in HCC. These findings help elucidate the diverse biological and pharmacological effects of MetF, particularly its influence on autophagy, as well as the potential of MetF as a therapeutic agent for diseases characterized by VDAC1 overexpression.

## Metformin Targets VDAC1 to Induce Autophagy in Cancer

Metformin, a common type 2 diabetes drug, is known for its glucose-lowering effects. The study used several cell lines and advanced techniques to investigate how Metformin induces cancer cell death. This experimental research included cell cultures and molecular analysis. They found that Metformin targets a mitochondrial protein called VDAC1. This interaction disrupts energy production and increases autophagy, leading to cancer cell death. The results showed that Metformin binds to VDAC1, reducing mitochondrial calcium and ATP levels, which activates autophagy and kills cancer cells. The researchers concluded that the ionic interaction of Metformin with VDAC1 is critical for its anticancer effects. Future studies could explore Metformin as a treatment for cancers with high VDAC1 expression. This summary was initially drafted using artificial intelligence, then revised and fact-checked by the author.

## Introduction

Metformin (MetF) is derived from guanidine, an active ingredient in French lilac. As a first-line treatment for type 2 diabetes, it is the most widely used drug throughout the world given its glucose-lowering effect, safety, oral availability and low price^1,2^. Specifically, MetF suppresses hepatic gluconeogenesis and improves insulin sensitivity. Hepatocellular carcinoma (HCC), the most common form of liver cancer, is closely associated with diabetes and is a leading cause of cancer-related mortality. A meta-analysis revealed that MetF reduced the risk of liver cancer by more than 60% in patients with type 2 diabetes^3^. It has been suggested that MetF inhibits mitochondrial respiratory complex 1 and ATP production^4^; however, the exact mechanism by which MetF regulates mitochondria is unclear.

The activation of AMPK, an important sensor that maintains cellular energy homeostasis, and the induction of autophagy trigger cell death in HCC^5,6^. Recent studies have focused on drug repositioning of MetF given its anticancer potential through the activation of AMPK signaling. Teng M. et al. reported that a low dose (30 μM) of MetF activates AMPK by targeting lysosomal PEN2 and that PEN2 is essential for MetF-induced glucose-lowering effects^7^. However, at concentrations sufficiently high to kill cancer cells, the anticancer targets of MetF and its mechanism of action have not been elucidated.

The high hydrophilicity of MetF, with its guanidine core structure, prevents the passive diffusion of the compound into cells; thus, a suitable carrier is required for drug transport^8^. Organic cation transporter 1 (OCT1/SLC22A1), which is expressed mainly in the liver, mediates the hepatic uptake of various positively charged hydrophilic compounds^9,10^. MetF exists in the cationic state at physiological pH and is an OCT1 substrate. The electrical properties of mitochondria are highly negative because of the flow of electrons along the electron transport chain (ETC) during ATP generation. During this process, protons are pumped out of the mitochondrial matrix, resulting in a negative potential ΔΨm (>−240 mV)^11^. This finding indicates that the positively charged MetF^12^ can be sufficiently targeted to mitochondria.

Here, we investigated the molecular mechanisms by which MetF activates autophagy-associated cell death by targeting mitochondria. Using an innovative label-free compound target identification platform called drug affinity responsive target stability (DARTS)-LC-MS/MS^13^, we demonstrated that MetF binds to the mitochondrial voltage-dependent anion channel 1 (VDAC1) of the mitochondrial proteome. VDAC1 maintains the structure of the mitochondria-associated ER membrane (MAM) by interacting with IP_3_R and GRP75^14^. The IP_3_R-GRP75-VDAC1 complex in MAMs is an important lineage for calcium transfer, and dysregulation of ER-mitochondrial Ca^2+^ exchange plays a role in promoting cancer hallmarks^15–17^. The positively charged natural compound MetF, which interacts with the anionic hotspot amino acid residue of VDAC1 (D9, E203), disrupts ER-mitochondria interactions and reduces Ca^2+^ influx into the mitochondria, thereby reducing ATP, activating the AMPK pathway and inducing autophagy-related tumor cell death. This study reveals a novel molecular mechanism for the anticancer activity of high-dose MetF, highlighting its interactions with a newly identified target protein, VDAC1, located on the mitochondrial membrane.

## Materials and methods

### Cell culture

HepG2, Huh-7, LX-2, HeLa and HEK293 cells were grown in DMEM (Thermo Fisher Scientific, Gibco™, Waltham, MA, USA) supplemented with 10% FBS (Gibco) and 1% antibiotics (Gibco). All the cell cultures were maintained at pH 7.4 in a humidified incubator at 37 °C under 5% CO_2_ in air.

### DARTS

HepG2 mitochondrial pellets were obtained via the Mitochondria Isolation Kit (89874, Thermo Fisher Scientific, Waltham, MA) and then lysed with 2% CHAPS in Tris-buffered saline to a final protein concentration of 1 mg/mL. All steps were performed on ice or at 4 °C to help prevent premature protein degradation. Protein samples were treated with MetF or distilled water for 3 h at 4 °C on a rocker and then incubated with or without pronase (10165921001, Roche, Germany) for 20 min at 25 °C. After the reaction, 6X SDS sample buffer was added to the sample, and the samples were heated at 100 °C. A portion of each sample was used for LC-MS/MS analysis.

### Sample preparation for LC-MS/MS analysis

DARTS samples of the in–solution molecular weight-based fractions were prepared for mass spectrometric analysis. The samples were reduced with 25 mM 1,4-dithiothreitol (3483-12–3, SigmaAldrich) for 30 min at 60 °C, alkylated with 25 mM iodoacetamide (114-48-9, SigmaAldrich) for 30 min at RT in the dark, and digested with trypsin (V528A, Promega, Madison, WA) overnight at 37 °C. The tryptic peptides were desalted using C18 cartridges and then dried.

### Immunoblotting

Soluble proteins were harvested from cells using SDS lysis buffer. Equal amounts of proteins were separated using 8% or 12.5% SDS-PAGE and transferred to polyvinylidene fluoride membranes. The blots were then blocked and immunolabeled with primary antibodies against β-actin (ab6276, Abcam, Cambridge, UK), AMPK (#2532, Cell Signaling Technology, Danvers, MA), phospho-AMPK (#2535, Cell Signaling Technology), β-tubulin (ab6276, Abcam), COX IV (#4844, Cell Signaling Technology), cytochrome C (ab133504, Abcam), GAPDH (#2118, Cell Signaling Technology), LC3B (ab48394, Abcam), mTOR (#4517, Cell Signaling Technology), phospho-mTOR (#2971, Cell Signaling Technology), Myc tag (M192-3, MBL Life Science, Japan), SQSTM1 (ab155686, Abcam), and VDAC1 (ab34726, Abcam) overnight at 4 °C. Immunolabeling was visualized using an enhanced chemiluminescence kit according to the manufacturer’s instructions. Images were quantified using Image Lab software (Bio-Rad Laboratories). All band intensities are proportional to the amount of target protein on the membrane within the linear range of detection.

### ATP-monitoring luminescence assay

To determine the cellular ATP levels, an ATPlite 1-step Luminescence Assay Kit (Perkin Elmer, Waltham, MA) was used according to the manufacturer’s instructions. The cells were seeded in black 96-well plates and incubated overnight. After drug treatment or knockdown, luminescence was measured using a Victor 3 multilabel plate reader (Perkin Elmer).

### Calcium assay

To monitor mitochondrial and cytosolic calcium, the cells were grown on chamber slides and incubated with 2 μM Rhod-2-AM (R1244, Invitrogen, Carlsbad, CA) or 2 μM Fluo-4-AM (F14201, Invitrogen) for 30 min in a 37 °C incubator. To validate the localization of Rhod-2-AM in the mitochondria, the cells were simultaneously treated with 100 nM MitoTracker (M7512, Invitrogen). The cells were then incubated in calcium-free Krebs-Ringer-HEPES buffer (pH 7.4) for an additional 30 min to de-esterify the dye. Images were captured using an LSM980 confocal microscope (Zeiss, Oberkochen, Germany). The signal intensity was quantified via ImageJ software.

### Proximity Ligation Assay

For in situ visualization of protein-protein interactions, the Duolink® In Situ Red Starter Kit (DUO92101, SigmaAldrich) was used according to the manufacturer’s instructions. The following primary antibodies were used: VAPB (66191-1-Ig, Proteintech) and PTPIP51 (20641-1-AP, Proteintech); IP_3_R-I (sc-271197, Santa Cruz Biotechnology, Dallas, TX) and VDAC1 (ab34726, Abcam); GRP75 (14887-1-AP, Proteintech) and VDAC1 (sc-390996, Santa Cruz Biotechnology). Images were captured using an LSM980 confocal microscope.

### Protein preparation for ensemble docking

Three X-ray crystal structures of human VDAC1 with PDB codes of 2JK4, 5XDO, and 6G6U were utilized. These three structures were considered because of the different configurational states, namely, monomeric, parallel dimeric, and antiparallel dimeric states, captured during X-ray crystallography (Supplementary Fig. 1). Before ensemble docking, all the structures were prepared using the “Protein Preparation Wizard” program, whereby missing loops and atoms were added and the protonation states of the ionizable residues were assigned at pH 7.4. The protein systems were also minimized using the conjugate gradient method in explicit water, applying the enhanced OPLS3 force field (OPLS3e)^18^.

### Ligand preparation for ensemble docking

To mimic the binding of MetF to VDAC1 at physiological pH, the protonation state of the ligand at pH 7.4 was determined using the pKa predictor available on the I-Lab™ browser operated by Advanced Chemistry Development, Inc. (ACD/Labs^®^) (Supplementary Fig. 2). Based on the pKa predictor, the monoprotonated form of MetF was preferred. The lowest-energy 3D conformations of the monoprotonated MetF were generated using Jaguar’s “QM Conformer and Tautomer Predictor” pipeline in Maestro^19^.

### Ensemble docking of MetF to VDAC1

To determine the potential binding hotspots of MetF on VDAC1, we performed ensemble docking using the Glide cross-docking workflow in Maestro version 11.7 (Schrödinger 2018-3, LLC, NY, USA). Prior to docking, the minimized VDAC1 structures were utilized to identify potential druggable sites with the SiteMap program in Maestro (Supplementary Fig. 3)^20,21^. Docking grids were generated on the basis of these druggable sites and later used for standard precision (SP) docking of the eight energy-minimized monoprotonated conformers of the MetF molecule (*vide supra*). To capture docking poses at different binding sites on VDAC1, cluster analysis based on structural interaction fingerprints (SIFt) developed by Deng et al.^22^ was performed.

### System preparation of the VDAC1-MetF complex

After performing ensemble docking and cluster analysis via SIFt, two binding configurations, namely, Poses 1 and 2 (Figs. 3a and 3b), were selected and prepared for molecular dynamics (MD) simulations. For each studied VDAC1-MetF configuration, the system was initially oriented such that the ion pore would be orthogonal to the least-squares best plane of the membrane bilayer. This was achieved using coordinates obtained from the PPM server of the Orientations of Proteins in Membrane database^23^. The resulting VDAC1-MetF model was submitted to the CHARMM-GUI Membrane Builder server to embed the system into a membrane bilayer comprising 1,2-dioleoyl-sn-glycero-3-phosphocholine (DOPC) and cholesterol (CHL) molecules at a ratio of 10:1, with 110 DOPC and 11 CHL molecules per leaflet^24,25^. The composition of the membrane bilayer is designed to approximately mimic the outer mitochondrial membrane of mammalian cells, which is composed of mainly phosphatidylcholines ( ~ 45%) and a small amount of CHLs ( ~ 4%)^26^. After the protein in the membrane bilayer was embedded, the system was solvated in TIP3P water with the minimum distance between the lipid molecules and the edge of the water box set to 22.5 Å (Fig. 3e). To create an environment close to physiological conditions, 39 K^+^ and 43 Cl^-^ ions were randomly added to the water box, resulting in a 0.15 M KCl solution. This helped to dissolve and neutralize the protein–membrane system. Because the two systems of interest are generated separately via the CHARMM-GUI server, the constitutions of these systems differ slightly. The overall dimensions of the two systems were approximately 100 Å by 100 Å by 93Å in the x, y, and z directions, respectively. The total number of atoms in each system was approximately 86000 atoms, with approximately 16000 water molecules.

### Computational alanine scanning mutagenesis

Residues contributing significantly to the binding of MetF were subjected to computational alanine scanning mutagenesis (CAS) using the *MMPBSA.py* program in AMBER 20. Two hundred snapshots from the last 10 ns of the 150 ns MD simulation, with 50 ps intervals between snapshots, were extracted for the calculation. As the name suggests, selected residues were mutated to alanine. Binding free energy calculations were performed for the mutated VDAC1-MetF complex and compared to those of the wild-type (WT) system. The difference in binding free energy between the WT and mutated (Mut) VDAC1-MetF complex (ΔΔG) was calculated to estimate the effect of mutation on binding ($\triangle \triangle G={\triangle G}_{{WT}}-\triangle {G}_{{Mut}}$). The more negative the ΔΔG is, the greater the destabilizing effect of the mutation on the VDAC1–MetF complex. This suggests the importance of the residue in MetF binding.

### Statistical analysis

All the data are presented as the means ± SEMs. The quantitative data were obtained from at least three independent experiments unless otherwise specified. Statistical analyses were performed using an unpaired, two-tailed Student’s t-test with GraphPad Prism (ver. 9 for Windows; GraphPad Software, Inc., San Diego, CA, USA). * indicates *P* < 0.05, ** indicates *P* < 0.01, *** indicates *P* < 0.001.

## Results

### MetF induces autophagy-induced cell death in HCC

**Fig. 1:**
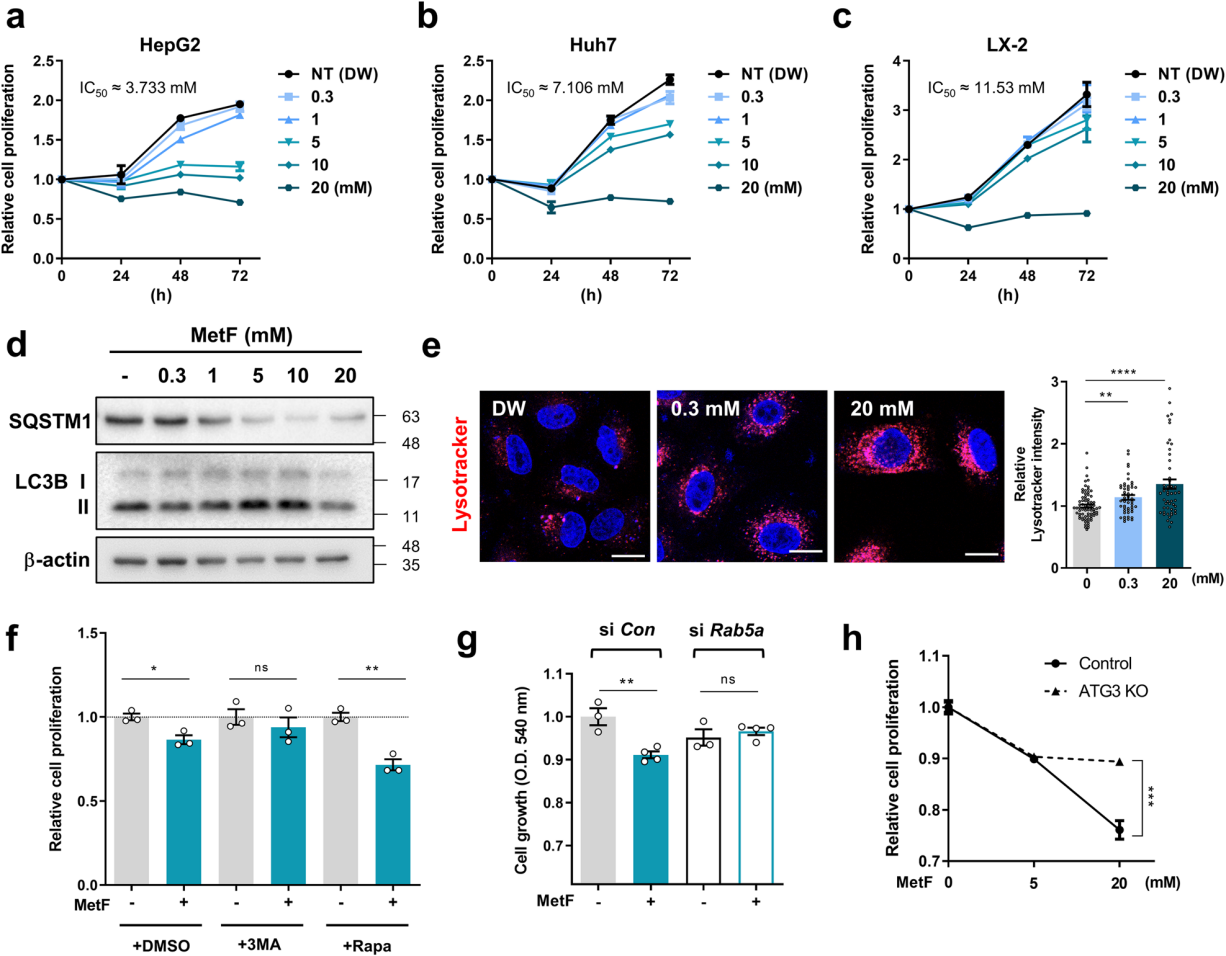
MetF inhibits hepatocellular carcinoma growth by inducing autophagy. **a**-**c** Effects of MetF treatment on the proliferation of HepG2, Huh7, and LX-2 cells. An MTT colorimetric assay was used to measure cell proliferation. **d** Western blot analysis of the levels of SQSTM1 and LC3 in HepG2 cells after MetF treatment (0.3–20 mM) for 24 h. **e** LysoTracker staining showed that MetF treatment induced lysosomal acidification in HepG2 cells. The cells were stained with LysoTracker Red, and the intensity was detected via confocal microscopy (scale bar: 20 μm). The plots represent lysosomal acidification signals in single cells. **f** HepG2 cell proliferation was measured after treatment with MetF (5 mM) using the MTT assay in the presence of 3-MA or Rapa (Rapamycin). **g** HepG2 cell proliferation was measured after Rab5a knockdown and treatment with MetF (5 mM), as determined by the MTT assay. **h** Cell proliferation was assessed in ATG3 knockout HeLa cells after MetF treatment. Statistical significance was evaluated using Student’s t-test (***P < 0.001, **P < 0.01, *P < 0.05).

To assess the impact of MetF on the inhibition of cell proliferation in HCC cell lines (HepG2 and Huh-7) and non-HCC human hepatic stellate cells (LX-2), the cells were treated with varying concentrations of MetF for 24–72 h. MTT assays revealed that MetF inhibited cell proliferation at lower concentrations in HCC than in normal liver cells (Fig. 1a-c). Analysis of the autophagic markers confirmed that MetF induced autophagy (Fig. 1d) and that lysosomal activity was increased after MetF treatment (Fig. 1e).

To investigate the potential role of autophagy in cell growth inhibition mediated by MetF, we examined the effects of co-treatment with an autophagy inhibitor (3-MA) or an autophagy inducer (Rapa). The inhibition of autophagy with 3-MA attenuated the suppressive effect of MetF on cell proliferation, whereas treatment with Rapa further enhanced cell growth inhibition (Fig. 1f). Next, we evaluated MetF activity in two cell types in which key components of the autophagy pathway were deleted. The inhibitory effect of MetF on cell growth was reduced in cells in which RAB5A, a key regulator of autophagy initiation, was knocked down (Fig. 1g, Supplementary Fig. 4a). Knockout of ATG3, an essential factor in autophagosome synthesis, alleviated the inhibitory effect of MetF on cell proliferation (Fig. 1h, Supplementary Fig. 4b). Taken together, these results suggest that autophagy contributes to the inhibition of HCC proliferation induced by MetF.

### Identification of the mitochondrial binding partner of MetF

DARTS is based on changes in resistance to proteolysis due to conformational changes induced when a compound of interest and proteins bind^13^. In our previous study, we used DARTS-LC-MS/MS to identify targets of autophagy-related compounds^27–30^. Using this technique, we identified the potential mitochondrial target proteins of unmodified MetF.

**Fig. 2:**
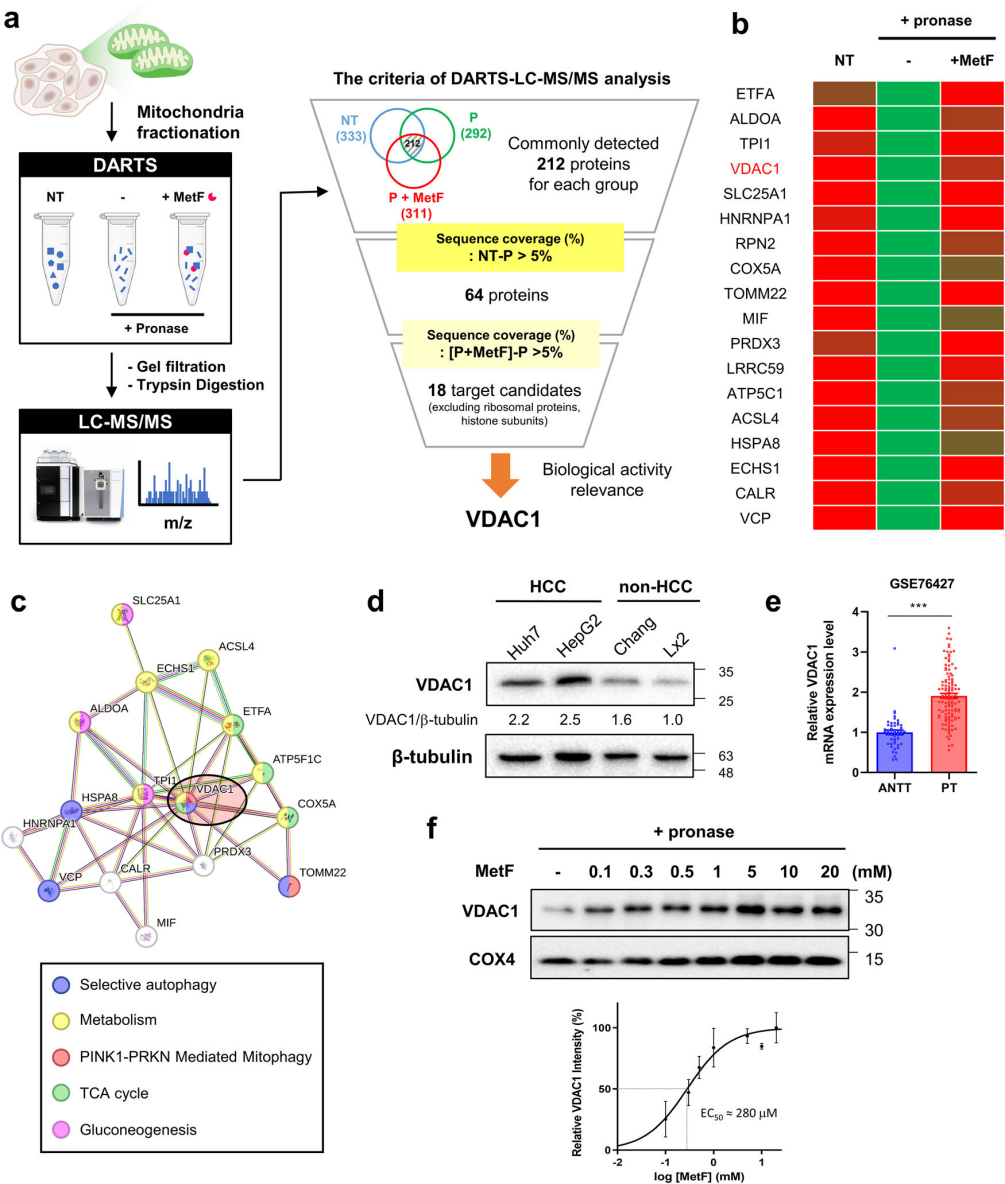
Target identification of MetF using DARTS-LC-MS/MS analysis. **a** Schematic representation of the label-free target identification procedure using drug affinity responsive target stability (DARTS). The mitochondrial proteins from the HepG2 cells were isolated and reacted with MetF for 1 h. The total protein pool was detected via LC-MS/MS analysis. **b** Heatmap of selected proteins with high sequence coverage resistant to protease degradation detected by DARTS-LC-MS/MS. **c** Functional analysis of candidate target proteins. **d** Immunoblot analysis of VDAC1 expression levels in liver cancer-derived and non-cancerous liver cells. **e** Differences in VDAC1 expression levels between adjacent non-tumor tissues (ANTTs) and primary tumors (PTs). The clinical significance of VDAC1 expression was analyzed in the HCC cohort (GSE76427). **f** Biophysical binding of different doses of MetF to VDAC1 was validated by DARTS. MetF increased the stability of VDAC1, which was degraded by pronase treatment (20 μg/mL, 20 min). The EC_50_ value was obtained via a logarithmic plot of the data. COX4 was used as a control to verify the mitochondrial protein fractions.

To focus on the effects of MetF on mitochondria, mitochondrial proteins were isolated from HepG2 cells. MetF was then reacted with the protein pool at a concentration of 300 μM for 1 h at 4 °C, and proteolysis of the proteome was performed by pronase (Fig. 2a). LC-MS/MS analysis was performed on the control group, the pronase-treated group, and the pronase- and MetF-treated groups. Among the total 212 proteins, 64 were degraded by more than 5% by pronase. Among these, 18 candidate target proteins resist pronase degradation because of their binding to MetF, resulting in an increase in sequence coverage of more than 5% (Fig. 2a, b). Reactome pathway analysis using the STRING database (https://string-db.org/) revealed that the candidate target proteins were involved in specific cellular processes, including selective autophagy, metabolism, PINK1–PRKN-mediated mitophagy, gluconeogenesis and the TCA cycle (Fig. 2c). To elucidate the molecular mechanisms underlying the effects of MetF on autophagy and cancer metabolism, VDAC1 was selected as a direct target. The sequence coverage of VDAC1 increased by 11.4% when VDAC1 was combined with MetF, which is a higher FC value than those obtained for other target candidates.

Next, we compared VDAC1 expression levels in HCC (Huh7 and HepG2) and normal liver cells (Chang, LX-2). As shown in Fig. 2d, VDAC1 was more highly expressed in HCC cells than in Chang and LX-2 cells. In addition, VDAC1 expression in HCC and normal tissues was also examined to determine whether a difference existed. RNA sequencing data, including data from 115 HCC tissue samples and 52 adjacent nontumor tissues (ANTTs), were obtained from the GEO database (http://www.ncbi.nlm.nih.gov/geo/). A 2-fold increase in VDAC1 expression was noted in primary HCC tumor tissues compared with adjacent nontumor tissues, highlighting its strong association with HCC (Fig. 2e).

To validate whether MetF binds to VDAC1, we monitored changes in the stability of VDAC1 to pronase as a function of the MetF concentration (Fig. 2f). The dose dependent curve allowed evaluation of the binding affinity of MetF and VDAC1. The half-maximal effective concentration (EC_50_) of ligand and protein binding was estimated to be approximately 280 μM. In addition, microscale thermophoresis (MST) assays revealed that MetF binds to the VDAC1 protein with a Kd value of 204 µM (Supplementary Fig. 5).

### Identification of hotspots on VDAC1 for MetF binding using computational alanine scanning (CAS) mutagenesis

**Fig. 3:**
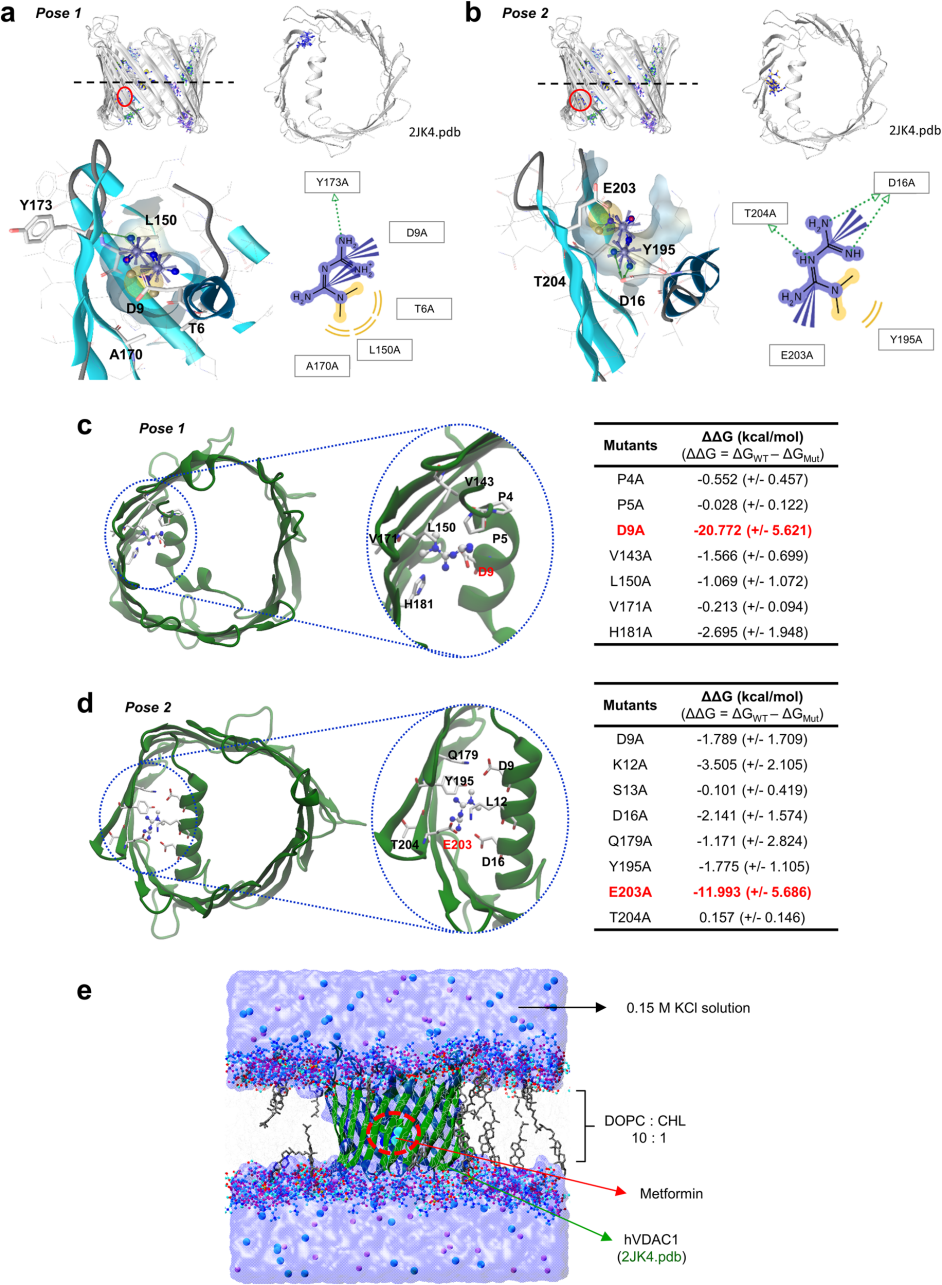
In silico analysis of the MetF binding site for VDAC1. **a** *(top)* Binding site of MetF in Pose 1. *(bottom)* 2D and 3D representations of the interactions formed between VDAC1 and MetF in Pose 1 generated using LigandScout 4.4. **b** *(top)* Binding site of MetF in Pose 2. *(bottom)* 2D and 3D representations of the interactions formed between VDAC1 and MetF in Pose 2 generated using LigandScout 4.4. In both poses (a and b), the MetF molecule binds near the N-terminal helix of VDAC1, closer to the inner membrane space, as indicated by the location of MetF below the dotted lines drawn across the center of the ion channel. The binding site of MetF (circled in blue) in the Pose 1 (**c**) or Pose 2 (**d**) configuration is illustrated. Residues with significant contributions to the binding of MetF in this region. **e** Schematic representation of the VDAC1-MetF complex embedded in 1,2-dioleoyl-sn-glycero-3-phosphocholine (DOPC) and cholesterol (CHL) molecules at a 10:1 ratio used for MD simulation. All systems constructed in this study for MD simulations and binding free energy calculations share a similar composition to that of this model.

Next, sites on VDAC1 with a high probability of MetF binding were investigated using computational analysis. On the basis of the results obtained via ensemble docking and cluster analysis via SIFt, two large clusters with the best average docking scores were selected for further analysis. From each cluster, the best docking pose based on docking scores was selected as a representative structure for further investigation, namely, Poses 1 (Fig. 3a) and 2 (Fig. 3b). These structures were equilibrated at 303.15 K and 1 atm using conventional MD simulation, and the last 10 ns of the 150 ns MD simulations were used to calculate the binding free energy of Poses 1 and 2. Per-residue energy decompositions were performed for the two selected docking poses to elucidate residues with significant contributions to the binding free energy of the VDAC1–MetF complex (Supplementary Fig. 6). These residues were then subjected to computational alanine scanning mutagenesis (CAS) to confirm their involvement in supporting the binding of MetF to VDAC1. For Pose 1, the following residues were considered for CAS: P4, P5, D9, V143, L150, V171, and H181 (Fig. 3c). For Pose 2, the following residues were considered: D9, L12, S13, D16, Q179, Y195, E203, and T204 (Fig. 3d). The ΔΔG values calculated for the residues in Poses 1 and 2 are shown in Fig. 3c and Fig. 3d, respectively.

On the basis of the CAS calculations performed, four hotspots were proposed to be critical for MetF binding to VDAC1. The CAS calculations performed for Pose 1 led to the identification of two possible binding hotspots, as shown by the significant destabilization of MetF binding upon mutation to alanine. These mutations are **D9A** and **H181A**, which resulted in ΔΔG values of −20.77 and −2.70 kcal/mol, respectively. On the other hand, the calculation performed for Pose 2 suggested three possible binding hotspots. One of the hotspots identified for Pose 2 was D9, which was also predicted by the CAS calculations performed for Pose 1. However, the D9A mutation in Pose 2 caused less destabilization of MetF binding than did Pose 1, with a reported ΔΔG of −1.79 kcal/mol. The other predicted hotspots for Pose 2 include **D16** and **E203**, which, when mutated to alanine, yielded ΔΔG values of −2.14 and −11.99 kcal/mol, respectively. Therefore, we propose two hotspots with the lowest ΔΔG at each position for further investigation via experimental mutation studies. These two residues are **D9 (Pose 1)** and **E203 (Pose 2)**. The schematic diagram in Fig. 3e shows a simulation of the VDAC1–MetF complex embedded in a membrane bilayer consisting of 1,2-dioleoyl-sn-glycero-3-phosphocholine (DOPC) and cholesterol (CHL) molecules.

### Validation of binding hotspots between MetF and VDAC1 in vitro

**Fig. 4:**
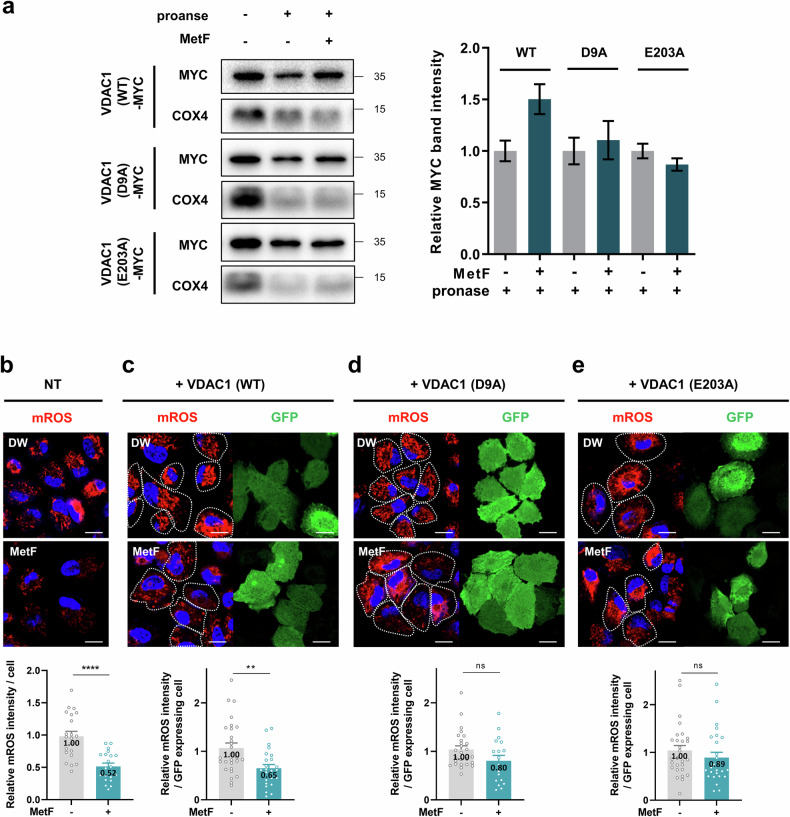
Validation of the binding between MetF and VDAC1. **a** HEK293 cells were transfected with MYC-VDAC1(WT), MYC-VDAC1(D9A) or MYC-VDAC1(E203A) vectors for 48 h each. The cells were then harvested, lysed and treated with MetF. Pronase treatment (5 μg/mL) was performed for 20 min. **b** Representative image and quantification of mROS in HepG2 cells after treatment with 5 mM MetF for 24 h. **c**–**e** HepG2 cells were transfected with the MYC-VDAC1 (WT), MYC-VDAC1 (D9A), or MYC-VDAC1 (E203A) vectors for 24 h. The cells were then treated with 5 mM MetF for an additional 24 h. Representative images captured by confocal microscopy and a graph quantifying mROS levels only in transfected cells (cells expressing GFP) are shown (scale bar: 20 μM). Statistical significance was evaluated with Student’s t-test (***P < 0.001, **P < 0.01, *P < 0.05).

Following the results of in silico modeling, we performed alanine mutagenesis on two residues, D9 and E203. The negative charge properties of aspartic acid and glutamic acid were lost by substitution with alanine. The resistance of VDAC1 to proteolysis by MetF was not restored in cells expressing VDAC1-D9A or VDAC1-E203A (Fig. 4a), suggesting that D9 and E203 are critical hotspot amino acid residues for the ionic interaction of MetF to bind to VDAC1.

Consistent with previous research indicating that MetF reduces mitochondrial ROS (mROS)^31^, we observed that MetF significantly inhibited mROS by approximately 50% in non-transfected (NT) HepG2 cells (Fig. 4b, Supplementary Fig. 7). Compared with those in normal cells, the mROS levels in VDAC1-overexpressing cells were increased by approximately 1.3-fold, and treatment with MetF resulted in a 35% reduction (Fig. 4c). The transfected cells were identified by GFP expression. However, mROS levels were reduced by only 20% and 11% in cells expressing the VDAC1 D9A and E203A mutants, respectively (Fig. 4d-e). Even with mutant VDAC1 transfection, MetF activity persists due to endogenous VDAC1 but is less effective than it is in WT VDAC1-transfected cells. Taken together, our data demonstrate that VDAC1 is a target protein that directly binds MetF and that the D9 and E203 residues are critical binding sites for MetF on VDAC1.

### MetF reduces ER-mitochondria interactions by targeting VDAC1 and increasing autophagy

**Fig. 5:**
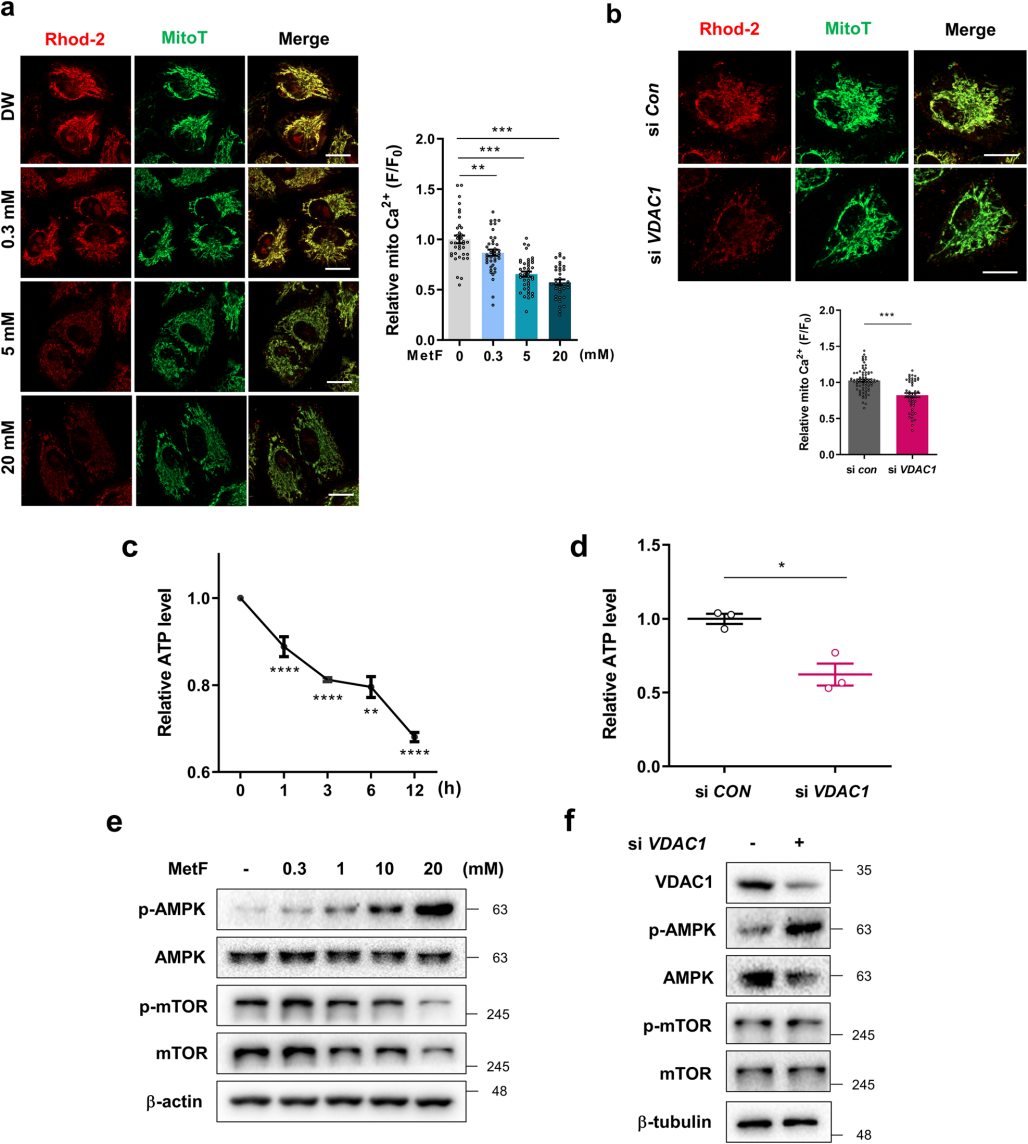
MetF treatment affects mitochondrial function and energy homeostasis. **a** HepG2 cells were treated with MetF for 4 h and stained with Rhod-2-AM, a red fluorescent dye, to detect mitochondrial calcium. Calcium signals were observed via confocal microscopy (scale bar: 20 μm), and the plots represent the calcium signals in single cells. **b** HepG2 cells were transfected with si *VDAC1* for 24 h and then stained with Rhod-2-AM. **c** MetF treatment decreased cellular ATP levels. **d** VDAC1 knockdown decreased cellular ATP levels. **e** Western blot analysis of the levels of AMPK-mTOR signaling factors in HepG2 cells after 24 h of MetF treatment. **f** Western blot analysis of the levels of AMPK-mTOR signaling factors after VDAC1 knockdown in HepG2 cells. Statistical significance was evaluated with Student’s t-test (***P < 0.001, **P < 0.01, *P < 0.05).

VDAC1 plays a role in regulating the flow of ions and molecules across the mitochondrial outer membrane, thereby affecting mitochondrial function. In HepG2 cells loaded with Rhod-2, a mitochondrial calcium indicator, MetF treatment reduced mitochondrial Ca^2+^ levels (Fig. 5a) and decreased ATP levels (Fig. 5c) in a dose-dependent manner. When VDAC1 was genetically knocked down, similar phenotypic changes were observed (Fig. 5b, d), suggesting a potential regulatory role of MetF in modulating mitochondrial function through its interaction with VDAC1. Given that an increase in the intracellular AMP:ATP ratio activates the AMPK pathway^32^, we examined the effect of MetF on AMPK–mTOR signaling. MetF treatment induces energy depletion, which leads to AMPK activation and the subsequent inhibition of mTOR. (Fig. 5e). As shown in Fig. 5f, when VDAC1 was knocked down, an increase in AMPK and a decrease in mTOR were also observed, suggesting that MetF directly binds to VDAC1 and regulates autophagy through the AMPK–mTOR signaling pathway.

The mitochondria-associated ER membrane (MAM), an interface between mitochondria and the ER, has garnered attention for its role in regulating of mitochondrial homeostasis, including Ca^2+^ and lipid transport, mitochondrial dynamics, autophagy, and apoptosis. Several protein interactions occur at the MAM; in particular, the IP_3_R-GRP75-VDAC1 complex is a key factor in strengthening the MAM and enabling Ca^2+^ exchange between the ER and mitochondria. The interaction of VAPB (an ER protein) with PTPIP51 (a mitochondrial protein) is a key component of the MAM and is involved in autophagy^33^. We next investigated whether MetF treatment alters the interaction between mitochondria and the ER by targeting VDAC1.

**Fig. 6:**
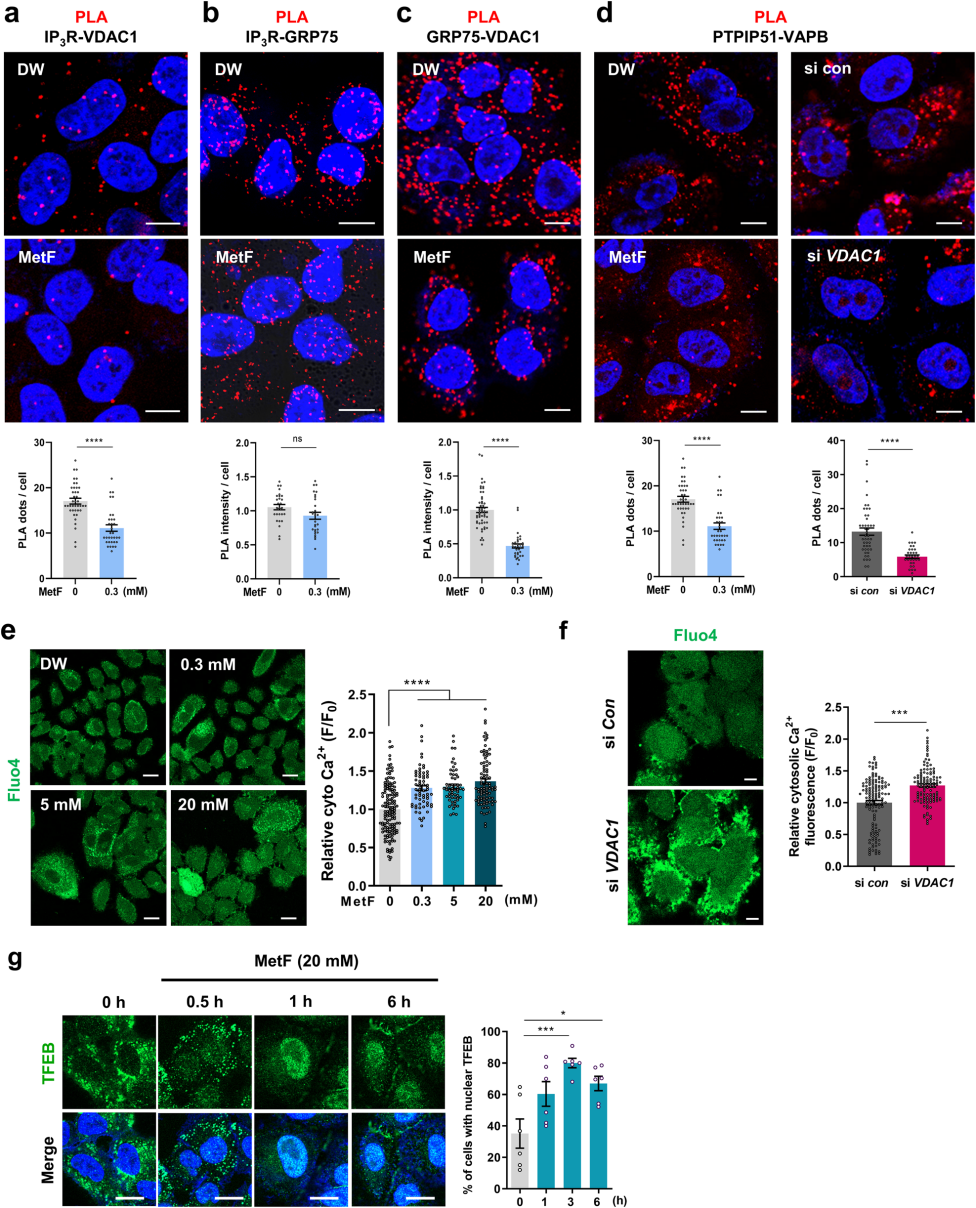
MetF reduces ER-mitochondria interactions and induces TFEB nuclear translocation. **a**–**c** Representative images of in situ proximity ligation assays (PLAs) of IP_3_R-VDAC1, IP_3_R-GRP75 and GRP75-VDAC1 in HepG2 cells after treatment with MetF (0.3 mM) for 4 h (scale bar: 10 μm). **d** Representative images of the in situ proximity ligation assay (PLA) between PTPIP51 and VAPB after treatment with MetF (0.3 mM) for 4 h or VDAC1 knockdown for 24 h in HepG2 cells (scale bar: 10 μm). Quantitative graph indicating the number of red dots per cell. **e** HepG2 cells were treated with MetF for 4 h and stained with Fluo-4-AM, a green fluorescent cytosolic calcium indicator. Calcium signals were visualized using confocal microscopy (scale bar: 20 μm), and the graphs represent calcium signals in single cells. **f** HepG2 cells were transfected with si *VDAC1* for 24 h and stained with Fluo-4-AM. **g** HepG2 cells were treated with MetF for 0–6 h, after which immunofluorescence staining was performed with a TFEB antibody (scale bar: 20 μm). Statistical significance was evaluated using Student’s t-tests (***P < 0.001, **P < 0.01, *P < 0.05).

The proximity ligation assay (PLA) results revealed that MetF treatment decreased the interaction between IP_3_R and VDAC1 (Fig. 6a). MetF also reduced the interaction between GRP75 and VDAC1 (Fig. 6c); however, interestingly, it did not significantly affect the interaction between IP_3_R and GRP75 (Fig. 6b). PLA analysis of VAPB and PTPIP51 in HepG2 cells revealed that MetF decreased organelle contacts in a dose-dependent manner (Fig. 6d). Consistent results were obtained after VDAC1 knockdown, with a reduced interaction noted between the two organelles (Fig. 6d), suggesting that the effect of MetF on MAMs is likely due to its direct interaction with VDAC1.

We hypothesized that as the distance between the ER and mitochondria increases, the calcium released from the ER is less likely to move to the mitochondria, resulting in an increase in the amount of calcium remaining in the cytosol. Cytosolic calcium levels measured with the Fluo-4 reagent were increased in MetF-treated cells (Fig. 6e) and VDAC1-depleted cells (Fig. 6f). Elevated cytosolic Ca^2+^ levels activate calcineurin, leading to the dephosphorylation of transcription factor EB (TFEB) and its nuclear translocation^34^. To further investigate the effect of cytosolic calcium upregulation by MetF binding to VDAC1, we examined whether MetF treatment regulates TFEB nuclear translocation. The ICC results indicated that MetF treatment led to the nuclear colocalization of TFEB (Fig. 6g). Given that TFEB is a master regulator of lysosomal biogenesis and autophagy induction, our results suggest that the increase in TFEB nuclear translocation may contribute to the autophagy-inducing effect of MetF treatment.

**Fig. 7:**
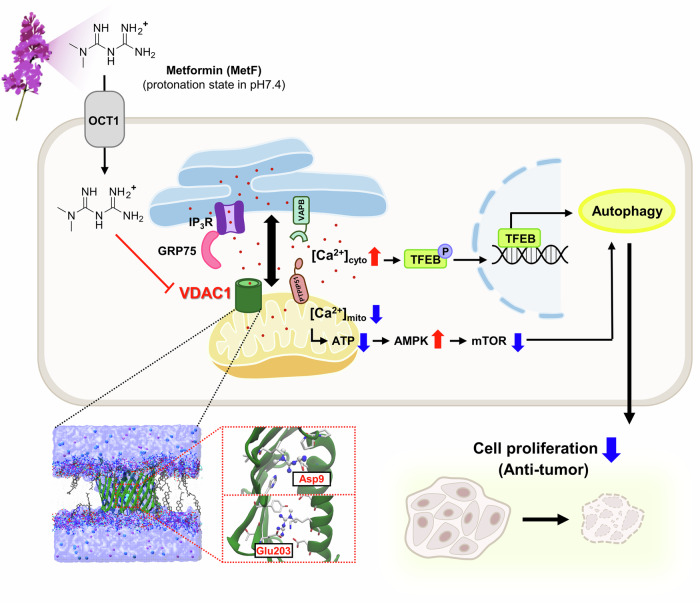
Schematic diagram showing the target proteins of MetF and its mechanism of action. MetF, which has a biguanide structure, carries a positive charge at pH 7.4 and thus crosses the cell membrane through channels such as OCT1. MetF binds to the mitochondrial protein VDAC1, with D9 and E203 of VDAC1 being critical sites for this interaction. MetF treatment increases the distance between the ER and mitochondria, leading to a decrease in mitochondrial calcium and ATP and an increase in cytosolic calcium. This activity induces autophagy, thereby promoting autophagy-dependent death in liver cancer cells.

Taken together, these findings demonstrated that VDAC1 knockdown mimicked the key activities induced by MetF, supporting the conclusion that VDAC1 is a biologically relevant target of MetF in that is involved in the regulation of autophagy in HCC (Fig. 7).

## Discussion

The mechanism of action of metformin (MetF), which has been widely used as a standard of care for diabetes for more than 60 years, remains to be fully elucidated. Interestingly, a growing body of literature suggests that the therapeutic potential of MetF may extend to various diseases, such as cancer, cardiovascular disease, obesity, aging, and autoimmune arthritis^35–37^. Although the effective concentration of MetF varies by cell line, it is generally accepted that AMPK is activated by millimolar concentrations of MetF. MetF has difficulty crossing hydrophobic cell membranes because of its hydrophilic, positively charged (at physiological pH) biguanide structure. Organic cation transporters allow MetF and other substances to cross cell membranes; however their transport capacity may not be sufficient to exhibit the physiological activity of MetF. This may explain why MetF has pharmacological effects even at high concentrations without causing significant toxicity. Typically, preclinical in vitro studies use drug concentrations that are 10 to 1000 times higher than those detected in patient plasma^38^. Given that the plasma concentration of MetF in patients taking MetF is approximately 30 μM^7,38^, the in vitro concentrations of MetF used in this study can be considered reasonable.

Recently, interesting studies have been conducted to identify the target proteins of MetF through affinity chromatography and elucidate its mechanism of action. Takahiro H. et al. revealed HMGB1 as a target protein of MetF^39^. They confirmed the binding to MetF at concentrations ranging from 1 to 100 mM and proposed a mechanism for its anti-inflammatory effects. Teng M. et al. revealed a mechanism by which low-dose (5–30 μM) MetF interacts with lysosomal PEN2 and increases AMPK without affecting the AMP/ATP ratio. However, this mechanism likely activates only a small amount of AMPK rather than resulting in the global activation of AMPK in the cell; thus, whether PEN2 regulation is sufficient to induce autophagy requires further investigation.

In this study, we focused on identifying new mitochondrial targets involved in the autophagy activity of MetF and obtained an overall profile of the entire mitochondrial proteome in response to label-free MetF (300 μM) after proteolysis via DARTS-LC-MS/MS. This method has been successfully used in many previous studies as a powerful method for target identification via unmodified compounds, and it is also effective for revealing multiple targets or off-target effects. VDAC1, a newly identified target of MetF, is a protein that is less susceptible to protease degradation due to MetF binding, suggesting that high doses of MetF interact with VDAC1 to induce conformational changes in the protein. In addition, abnormal VDAC1 expression is linked to several diseases^40^, and its high expression in HCC indicates that it could be a potential target for MetF-induced autophagy-related cell death.

We also predicted the critical binding site of MetF to VDAC1 using in silico modeling. Computational alanine scanning mutagenesis calculations revealed that D9 and E203 are key residues involved in the interaction of MetF with VDAC1. When D9 and E203 were mutated to alanine, significant destabilization of VDAC1–MetF binding was detected. Interestingly, the side chains of aspartic acid and glutamic acid are negatively charged as carboxylate groups with a pKa low enough to lose a proton, whereas MetF has a positive charge under most physiological conditions due to the intrinsically high pKa of the guanidine moiety. Electrostatic interactions, in which the negatively charged VDAC1 surface attracts the positively charged MetF, contribute to stronger binding interactions. In addition, we experimentally demonstrated that the VDAC1-D9A and VDAC1-E203A mutant proteins do not interact with MetF via DARTS in vitro, supporting the in silico predictions.

We further examined the reduced mitochondrial calcium, ROS, and ATP levels in VDAC1-knockdown cells, which could be rescued by re-expressing VDAC1 (WT, D9A or E203A) (Supplementary Fig. 8). In silico structural simulation results indicate that these mutations do not induce substantial changes in the cylindrical structure of VDAC1, even when the D9 or E203 residues are replaced by alanine (Supplementary Fig. 9). Therefore, these mutations do not appear to significantly affect VDAC1 function or trafficking. However, the lack of MetF activity in cells expressing the mutant VDAC1 suggests that the D9 and E203 residues play critical roles in MetF binding.

AMPK regulates several metabolic pathways, such as those involved in diabetes, hyperglycemia and obesity^41,42^; thus, the beneficial effects of MetF are largely due to AMPK activation. We observed that VDAC1 knockdown decreased the amount of ATP and upregulated AMPK and autophagy, mimicking the findings in MetF-treated cells. Our previous study also demonstrated the relationship between VDAC1 and the AMPK pathway; the antidepressant drug sertraline effectively reduces tau protein-induced toxicity through AMPK-dependent autophagy by targeting VDAC1^27^. Unlike MetF, the S196 site of VDAC1 is a key amino acid for sertraline binding. In that paper, we did not consider how VDAC1 is related to mitochondrial ATP levels. However, in the current study, we present a novel mechanism by which the modulation of VDAC1 with MetF reduces mitochondrial ATP. Calcium moves from the ER to the mitochondria via the IP_3_R-GRP75-VDAC1 complex in the MAM. Moreover, the MAM is attracting attention as a platform for regulating intracellular homeostasis. MetF binding to VDAC1 may reduce the interaction between the ER and mitochondria, leading to a decrease in calcium influx into the mitochondria. In addition, VDAC1–MetF binding may contribute to the regulation of ATP levels, AMPK signaling, and cytosolic Ca^2+^-mediated autophagy.

In conclusion, we demonstrated that MetF participates in autophagy by directly binding to the mitochondrial membrane protein VDAC1, enhancing the AMPK signaling pathway, and inhibiting ER-mitochondria interactions (Fig. 7). This study newly proposes that the ionic attraction between MetF(+) and VDAC1(-) offers a new perspective on how MetF regulates mitochondrial function. By highlighting the important role of VDAC1 in the AMPK-mediated autophagy-related cell death process, MetF could be explored as a potential autophagy regulator in diseases characterized by high VDAC1 expression, including HCC.

## Supplementary information

Supplementary Information
