# Supplementary material for: The anticancer effect of metformin targets VDAC1 via ER-mitochondria interactions-mediated autophagy in HCC
Source: Exp Mol Med. 2024 Dec 3;56(12):2714–25. doi: 10.1038/s12276-024-01357-1 (PMC11671597; doi:10.1038/s12276-024-01357-1)
Supplement: Supplementary file 1 — Supplementary Information [file 12276_2024_1357_MOESM1_ESM.pdf]

SUPPLEMENTARY INFORMATION

The anticancer effect of metformin targets VDAC1 via ER-mitochondria interactions-mediated autophagy in HCC

Minjeong Ko[1,5], Jiho Kim[1,5], Raudah Lazim[2,5], Ju Yeon Lee[3,4], Jin Young Kim[3,4], Vijayakumar Gosu[2], Yoonji Lee[2], Sun Choi[2,*] and Ho Jeong Kwon[1,*]

[1] *Chemical Genomics Leader Research Laboratory, Department of Biotechnology, College of Life Science and Biotechnology, Yonsei University, Seoul 03722, Republic of Korea.*
[2] *Global AI Drug Discovery Center, College of Pharmacy and Graduate School of Pharmaceutical Sciences, Ewha Womans University, Seoul 03760, Republic of Korea*
[3] *Research Center of Bioconvergence Analysis, Korea Basic Science Institute, Ochang 28119, Republic of Korea.*
[4] *Critical Diseases Diagnostics Convergence Research Center, Korea Research Institute of Bioscience and Biotechnology, Daejeon 34141, Republic of Korea*
[5] *These authors contributed equally to this work*

[*]**Co-Corresponding Authors**:

Prof. Ho Jeong Kwon: E-mail: kwonhj@yonsei.ac.kr. Tel: +82-2-2123-5883

Prof. Sun Choi: E-mail: sunchoi@ewha.ac.kr Tel: +82-2-3277-4503

SUPPLEMENTARY MATERIALS AND METHODS

Cell proliferation assay

HepG2 and Huh-7 were seeded in 96-well plates at 3,000 cells/well and incubated overnight. Metformin hydrochloride (1,1-Dimethylbiguanide hydrochloride) (D150959, Sigma-Aldrich, St.Louis, MO) was added to the cells to determine the effect on cell proliferation. Cells were grown from 0 to 72 h, and growth was analyzed by the 3-(4,5-dimethylthiazol-2-yl)-2,5-diphenyl tetrazolium bromide (MTT) (0793, VWR International, Radnor, PA) colorimetric assay. MTT formazan was dissolved in DMSO in each well, and the absorbance at 540 nm was read using a microplate reader.

Transfection

For gene knockdown studies, cells were transfected with the 50 nM siVDAC1 (L-019764-00-0005, Dharmacon, Lafayette, CO), siRAB5A (004009-00-0005, Dharmacon) for 24 h using Lipofectamine^{TM} RNAiMAX transfection reagent (Invitrogen) according to the manufacturer's instructions. A VDAC1 (Myc-DDK-tagged) human ORF Clone (RC209949, OriGene, Rockville, MD) was used for VDAC1 mutant vector generation. Cells were transfected with VDAC1 plasmid using Lipofectamine^{TM} 3000 transfection reagent (Invitrogen) according to the manufacturer's instructions.

NanoLC-ESI-MSMS analysis

The digested peptides were dissolved in mobile phase A and analyzed using an LC-MS/MS system consisting of a Nano Acquity UPLC system (Waters, Milford, MA) and an LTQ Orbitrap Elite mass spectrometer (Thermo Fisher Scientific) equipped with a nano-electrospray source. An autosampler was used to load 5-µL aliquots of the peptide solutions into a C18 trap column of i.d. 180 µm, length 20 mm, and particle size 5 µm (Waters). The peptides were

desalted and concentrated on the trap column for 10 min at a flow rate of 5 µL/min. The trapped peptides were then back-flushed and separated on a homemade microcapillary C18 column of i.d. 100 µm and length 200 mm (Aqua; particle size 3 µm, 125 Å). The mobile phases were composed of 100% water (A) and 100% acetonitrile (ACN) (B), each containing 0.1% formic acid. The LC gradient began with 5% mobile phase B and maintained for 15 min. The mobile phase B was linearly ramped to 15% for 5 min, 50% for 75 min, and 95% for 1 min. Then, 95% mobile phase B was maintained for 13 min before descending to 5% B for another 1 min. The column was re-equilibrated with 5% B for 10 min before the next run. The voltage applied to produce the electrospray was 2.2 kV. During the chromatographic separation, the LTQ Orbitrap Elite was operated in data-dependent mode. MS data were acquired using the following parameters: full scans were acquired in the Orbitrap at a resolution of 60,000 with 400 to 2000 m/z range for each sample; ten data-dependent collision-induced dissociation (CID) MS/MS scans were acquired per full scan; CID scans were acquired in a linear trap quadrupole (LTQ) with 20 ms activation times performed for each sample; 35% normalized collision energy (NCE) was used in CID; and 2 Da isolation window for MS/MS fragmentation was applied. Previously fragmented ions were excluded for 60 sec.

Protein identification

The Integrated Proteomics Pipeline using built-in search engines was utilized for data analysis with Uniprot Human protein database (released on December 04, 2014). The reversed sequences of all proteins were appended into the database for calculation of false discovery rate (FDR). ProLucid[1] was used to identify the peptides, a precursor mass error of 5 ppm, and a fragment ion mass error of 800 ppm. Trypsin was selected as the enzyme, with two potential missed cleavages. Carbamidomethylation at cysteine and oxidation at methionine were chosen as static and variable modifications respectively. The output data

files were filtered and sorted to compose the protein list using the DTASelect (The Scripps Research Institute, USA) with two and more peptides assignments for a protein identification and a false positive rate less than 0.05 [2].

Ligand preparation for ensemble docking

Three tautomers of the mono-protonated MetF were initially generated and single point calculations were performed using the parametric model number 3 (PM3) method. Conformational search was performed for the resulting three structures using the OPLS2005 force field to afford eight lowest-energy structures that were also subjected to another round of semi-empirical calculations. To further optimise these structures, two stages of quantum mechanics (QM) calculations were performed in continuum solvent. The solvation effect was reflected in the QM calculations by solving the Poisson Boltzmann equation to obtain a layer of discrete charges on the molecular surface that simulate the continuum boundary between the molecule and their surrounding environment. Geometry optimisation was performed for each configuration at B3LYP-D3/LACVP** level of theory. These structures were subsequently subjected to single point calculations at M06-2X/ cc-pVTZ(-f) level of theory to obtain the final conformers used for ensemble docking.

Equilibration of the VDAC1-MetF model using conventional MD simulation

Conventional MD simulations were performed for the two aforementioned prepared VDAC1-MetF systems using the Amber 20 simulation package[3]. VDAC1 was parameterised using the AMBER ff19SB force field[4] while the DOPC/CHL membrane bilayer was parameterised using the LIPID14 force field[5]. The parameters of the MetF molecule were generated using the antechamber suite of AmberTools 20.15[3,6]. The AM1-BCC charge model was used to compute the partial charges of MetF and force field parameters for MetF were obtained from the general Amber force field (gaff)[3,7]. Periodic boundary was applied for all simulations. During the

simulation, long-range electrostatic interactions were calculated using the particle mesh Ewald method and non-bonded interactions were truncated at 12 Å[8]. All bonds involving hydrogen atoms were constrained using the SHAKE algorithm[9].

Before conducting the MD simulation, a three-step energy minimisation was performed to relax the entire system in the following order of execution: (1) minimisation of solvent only, (2) minimisation of solvent and membrane bilayer, and (3) minimisation of the whole system. For all three steps, the minimisation was started using the steepest descent method for 5000 steps and thereafter for 5000 steps using the conjugate gradient method. After energy minimisation, the system was gradually heated from 10 K to 303.15 K over 100 ps using the Langevin thermostat under a canonical ensemble with a collision frequency of 1.0 ps^{-1}. During heating, weak harmonic restraints of 10 kcal/mol/$Å^2$ was imposed on the protein and membrane bilayer to prevent large fluctuations during temperature scaling. Equilibration of the system were subsequently conducted in the isothermal-isobaric ensemble for 5 ns at constant temperature and pressure of 303.15 K and 1 atm respectively prior to the production run which was conducted for 150 ns. For all simulations, an integration time step of 2 fs was used.

Binding free energy calculations of VDAC1-MetF complex

To compute the binding free energies of the VDAC1-MetF complex in selected binding configurations harnessed through ensemble docking, we have utilised the *MMPBSA.py* program implemented in AMBER 20 to conduct end-state free energy calculations[3,10]. 1000 snapshots were extracted from the last 10 ns of the 150 ns production MD simulation (*vide supra*), with equal intervals of 10 ps between snapshots, for binding free energy calculations using the molecular mechanics generalized Born surface area (MM-GBSA) method. This method evaluates the binding free energy (ΔG_{bind}) of VDAC1-MetF complex by calculating the free energy difference between the bound and unbound states of VDAC1-MetF complex

(Equation 1)[11]. In this study, we also evaluated the energy contribution of individual residues towards the binding of MetF by conducting per-residue energy decomposition. For this analysis, only residues within 5 Å of the MetF molecule were considered. During per-residue energy decomposition analysis, ΔG_{bind} was segregated into three distinct energy terms (Equation 2), namely (i) the gas-phase molecular mechanics (MM) energy (ΔE_{MM}) which comprised of van der Waals energies (ΔE_{vdw}), electrostatic energies (ΔE_{elec}) and internal energies (ΔE_{int}) (Equation 3), (ii) the solvation free energy (ΔG_{sol}) which aggregates the electrostatic solvation energy (ΔG_{GB}) and the non-polar contributions (ΔG_{SA}) between the solute and the continuum solvent (Equation 4), and (iii) the configurational entropy contributed by the binding of a ligand to the protein ($-T\Delta S$)[12]. For this study, calculation of the configurational entropy was not conducted as the calculation often provides inaccurate approximations for large systems despite being computationally costly. Furthermore, no comparisons were made between different systems in this study.

$$\Delta G_{bind} = G_{complex} - (G_{protein} + G_{ligand}) \quad (1)$$

$$\Delta G_{bind} = \Delta E_{MM} + \Delta G_{sol} - T\Delta S \quad (2)$$

$$\Delta E_{MM} = \Delta E_{vdw} + \Delta E_{elec} + \Delta E_{int} \quad (3)$$

$$\Delta G_{sol} = \Delta G_{GB} + \Delta G_{SA} \quad (4)$$

SUPPLEMENTARY FIGURES

Supplementary Figure 1.

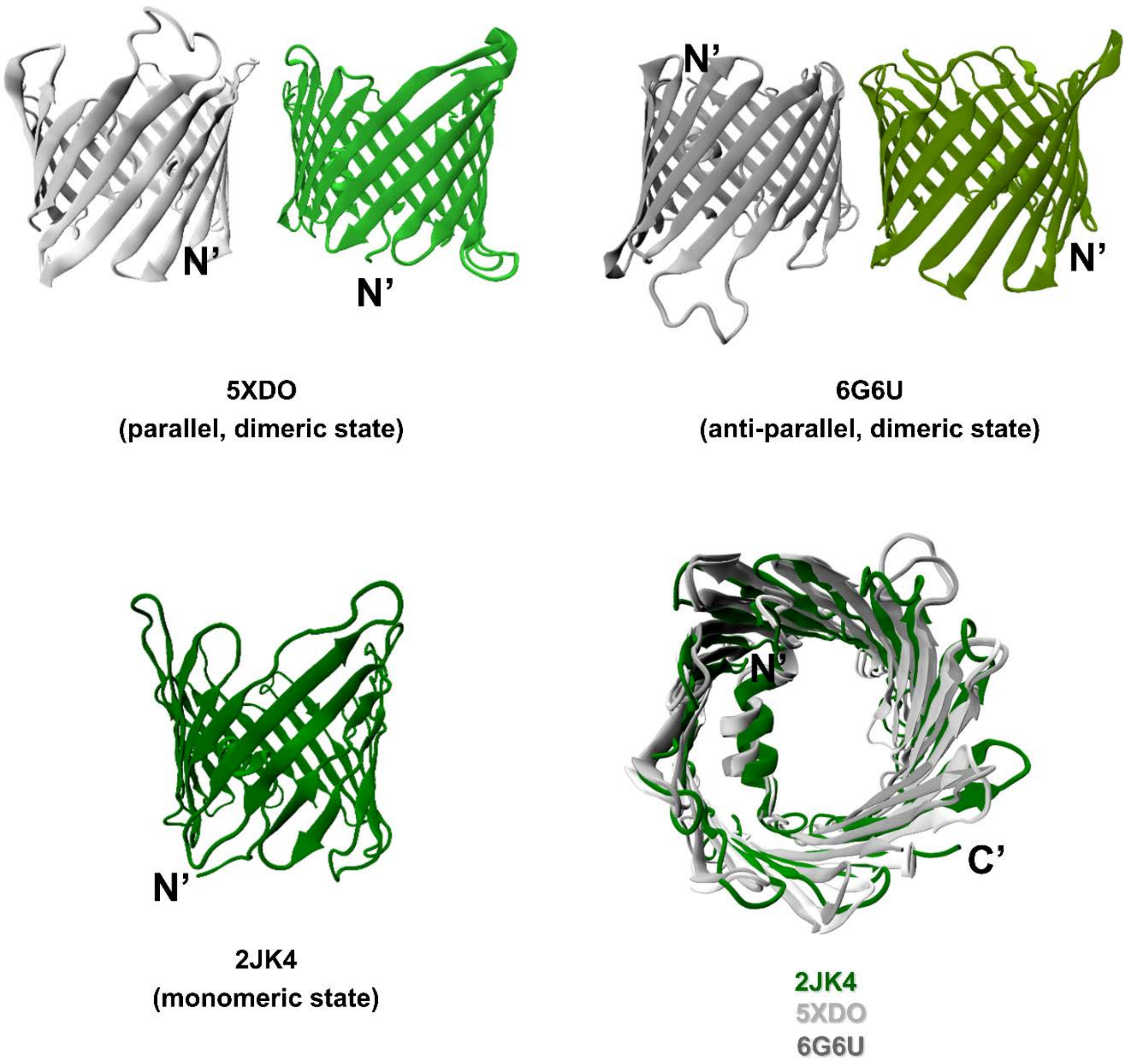

Three-dimensional x-ray crystal structures of human VDAC1 (PDB codes: 5XDO, 6G6U, and 2JK4) solved in three different configurations, namely monomeric, parallel dimeric, and anti-parallel dimeric states. Overlay of the monomers of each crystal structure is shown at the bottom righthand corner of the figure.

Supplementary Figure 2.

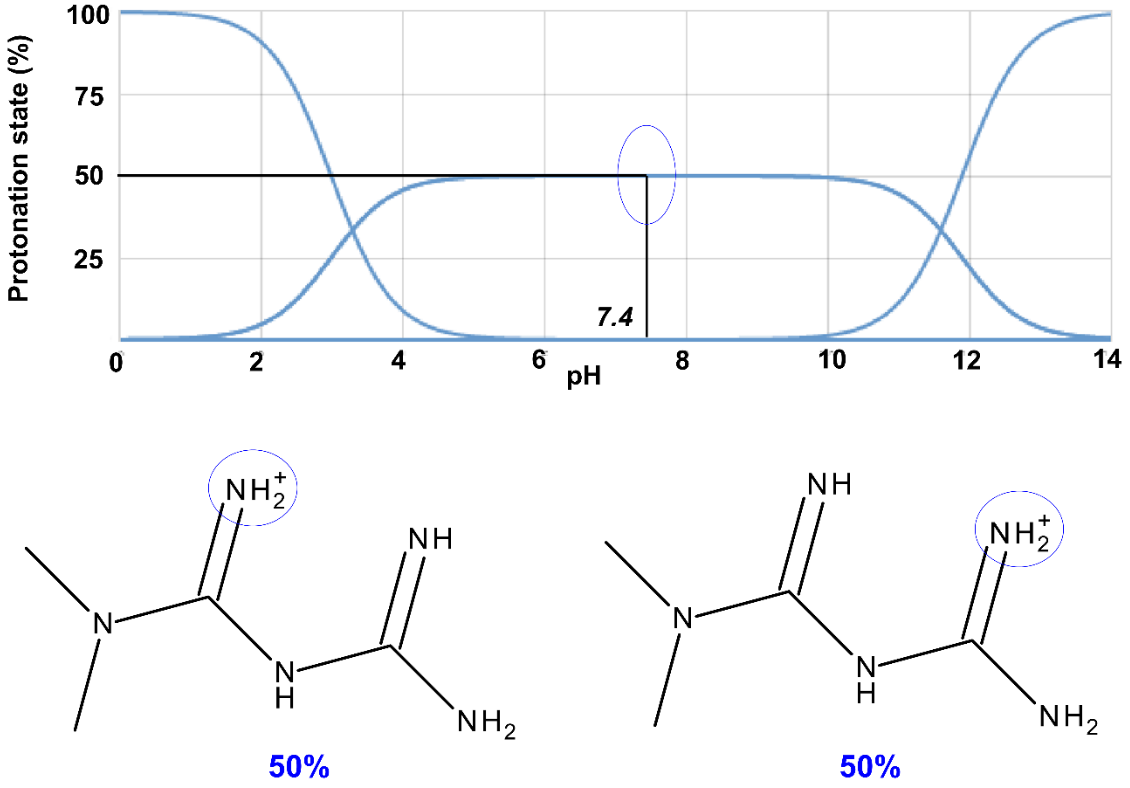

Plot of the population of protonation states versus pH acquired using I-Lab™ browser of ACD/Labs®. Depending on the pH, metformin can assume three protonation states, namely neutral, mono-protonated, and di-protonated. These states are represented by the three blue lines in the graph above. At pH 7.4, the mono-protonated forms of metformin are preferred with protonation occurring on either one of the two amine groups circled in blue.

Supplementary Figure 3.

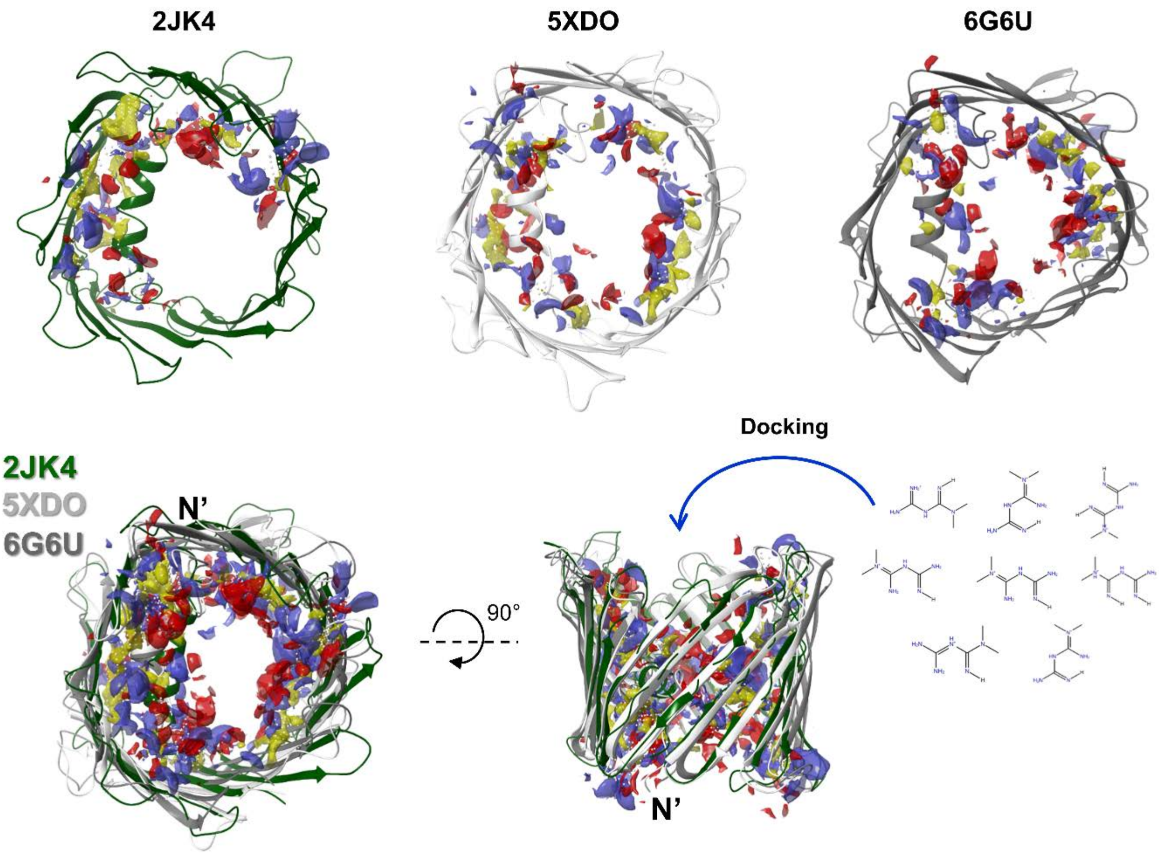

(*top*) Druggable sites of each VDAC1 crystal structures were determined using the SiteMap program. Blue patches correspond to hydrogen bond donor maps, red patches correspond to hydrogen bond acceptor maps, and yellow patches correspond to the hydrophobic maps on VDAC1. (*bottom*) The overlay of the three systems and their predicted druggable sites are also shown.

Supplementary Figure 4.

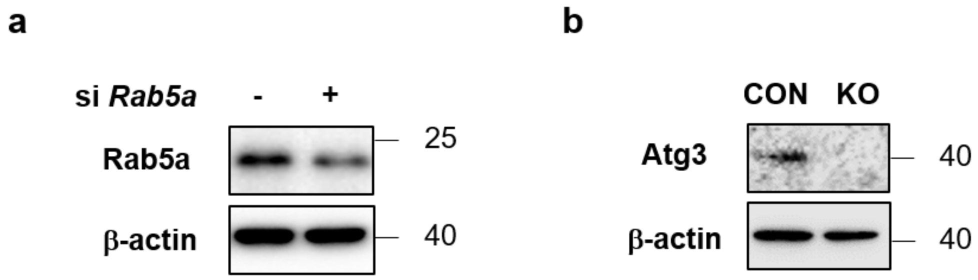

(a) Western blot analysis was performed to confirm the knockdown of Rab5a following 24 hours of siRNA treatment. **(b)** Western blot analysis was performed to confirm the knockout of Atg3

Supplementary Figure 5.

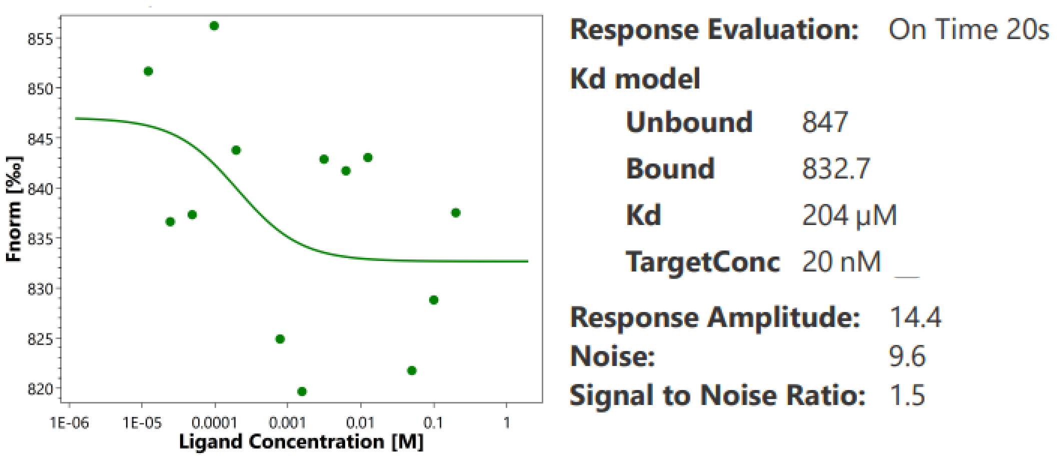

The MST-based binding affinity (Kd) of Metf to VDAC1 was measured using 20 nM of the recombinant human VDAC1 protein (abcam, Cambrideg, UK) labelled with the Monolith Protein Lableing Kit RED-NHS 2nd Generation (Nanotemper) and 200 mM Metf stock solution in Monolith X instrument (Nanotemper, Munich, Germany). Excitation color and auto-detected power were setted to Pico-RED and 13%, respectively. The MST-based binding affinity (Kd) of Metf to VDAC1 was 204 μM.

Supplementary Figure 6.

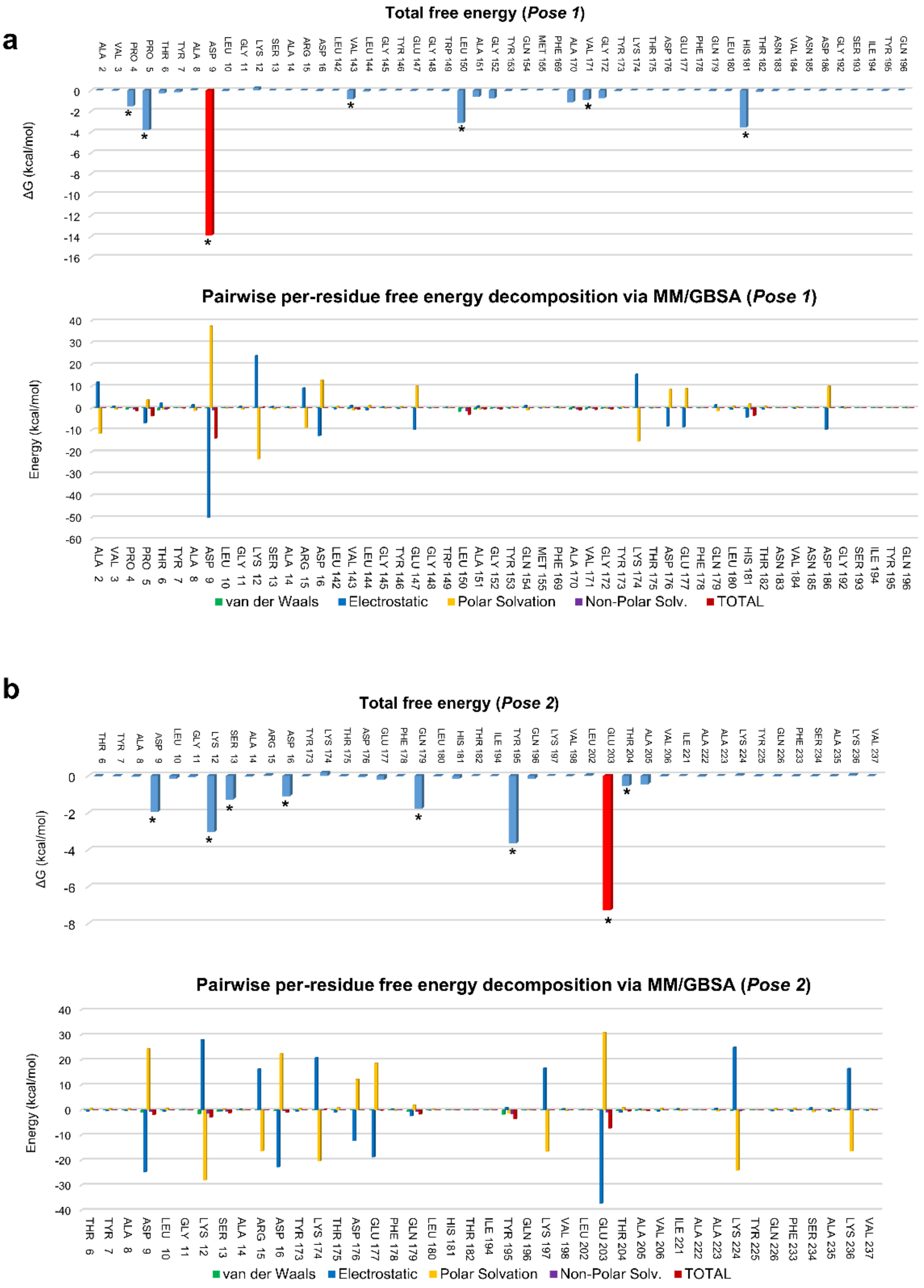

Total free energy and per-residue energy decomposition analysis of Pose 1(**a**) and Pose 2 (**b**). These residues (denoted by asterisks in the plot in Fig 3c,d) contribute significantly to the binding free energy of the VDAC1-Metformin complex.

Supplementary Figure 7.

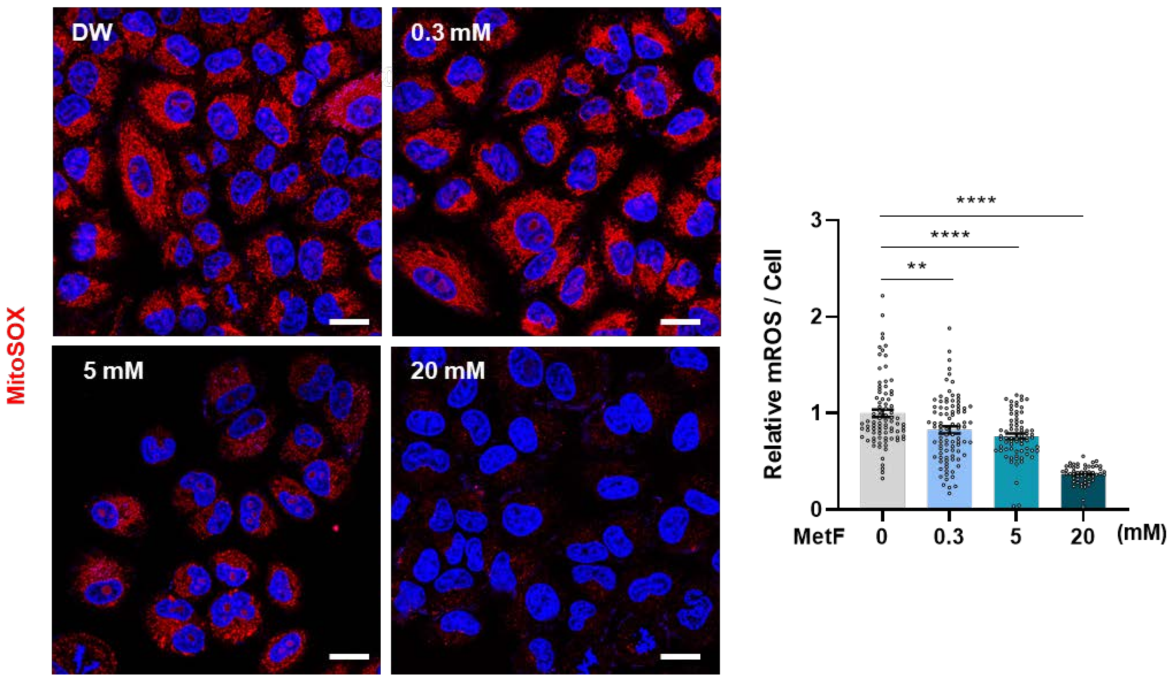

HepG2 cells were treated with MetF for 12 hours, followed by mROS evaluation using MitoSOX staining. Mitochondrial ROS signals were detected by confocal microscopy (scale bar: 20 μM).

Supplementary Figure 8.

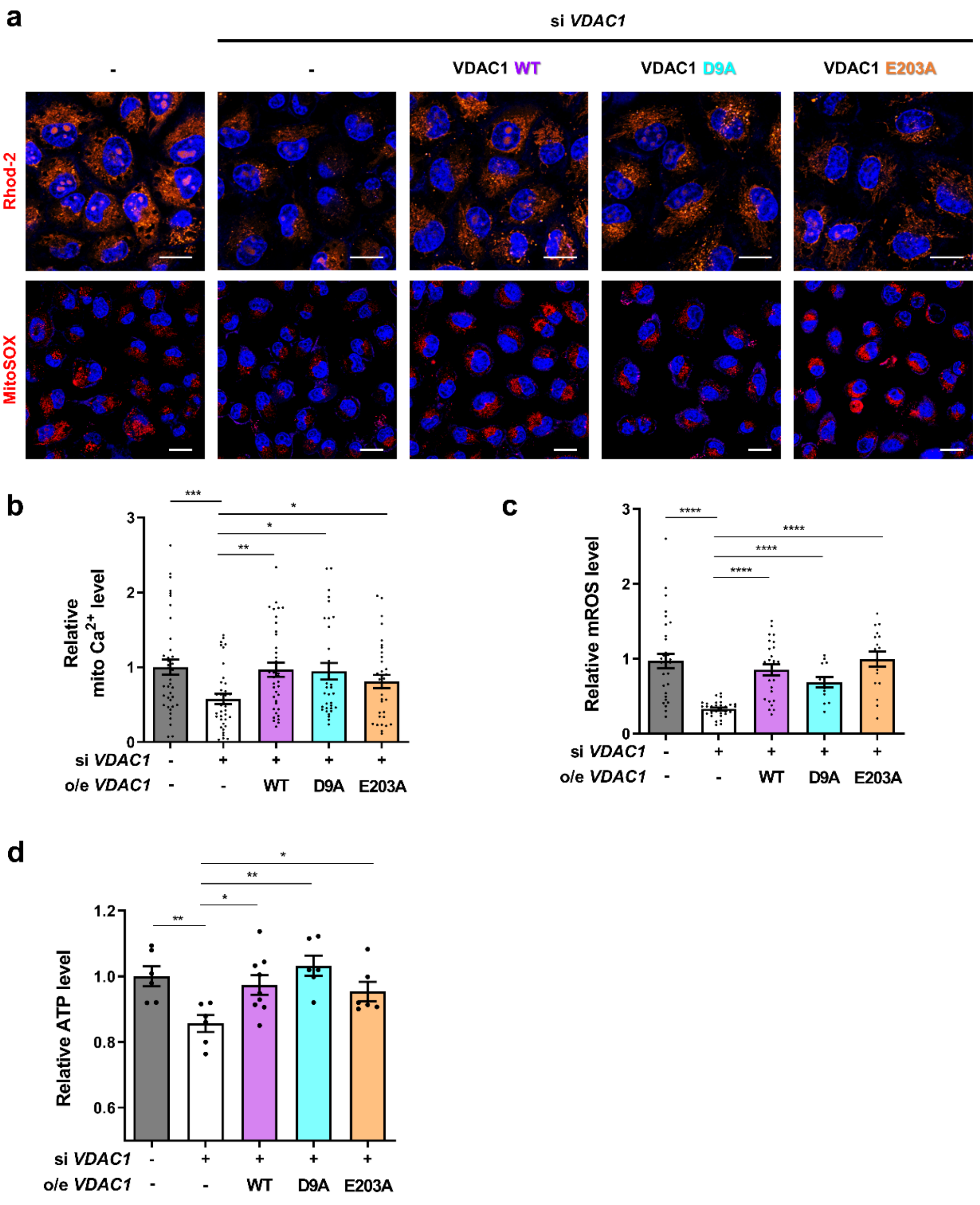

HepG2 cells were treated with si*VDAC1* (20 nM) to knockdown VDAC1 for 24 hours. Following this, cells were further incubated with VDAC1 (WT or D9A or E203A) clones for an additional 24 hours. Mitochondrial calcium (**a,b**), mitochondrial ROS (**a,c**), and ATP (**d**) levels were then measured (scale bar: 20 µM).

Supplementary Figure 9.

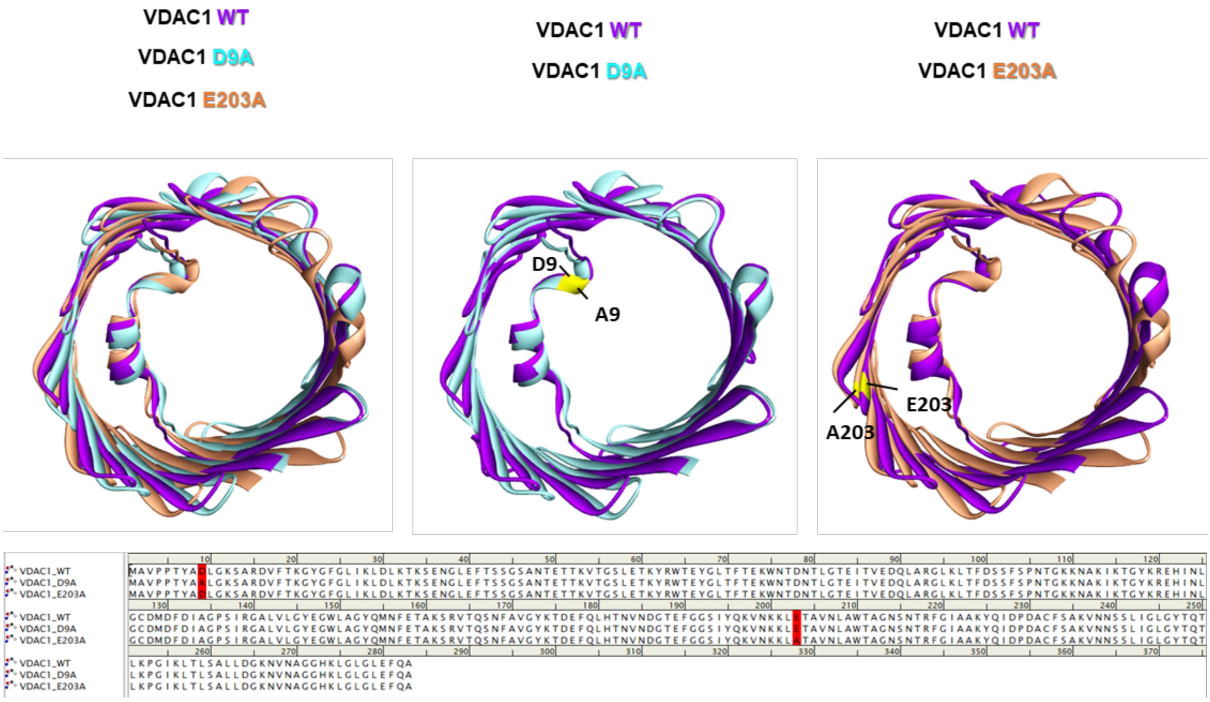

Structures of VDAC1 WT, VDAC1 D9A, and VDAC1 E203A generated using ColabFold v1.5.5: AlphaFold2 with MMseqs2.

SUPPLEMENTARY REFERENCES

1. Carvalho, P. C., et al. YADA: a tool for taking the most out of high-resolution spectra. *Bioinformatics.* **25**, 2734-2736 (2009).
2. Tabb, D. L., McDonald, W. H., and Yates, J. R. DTASelect and Contrast: tools for assembling and comparing protein identifications from shotgun proteomics. *Journal of Proteome Research.* **1**, 21-26 (2002).
3. Case, D. A., et al. (2020) *Amber 2020*, University of California, San Francisco
4. Tian, C., et al. ff19SB: Amino-acid-specific protein backbone parameters trained against quantum mechanics energy surfaces in solution. *J Chem Theory Comput.* **16**, 528-552 (2019).
5. Dickson, C. J., et al. Lipid14: the amber lipid force field. *J Chem Theory Comput.* **10**, 865-879 (2014).
6. Wang, J., Wang, W., Kollman, P. A., and Case, D. A. Automatic atom type and bond type perception in molecular mechanical calculations. *J Mol Graph Model.* **25**, 247-260 (2006).
7. Jakalian, A., Jack, D. B., and Bayly, C. I. Fast, efficient generation of high-quality atomic charges. AM1-BCC model: II. Parameterization and validation. *J Comput Chem.* **23**, 1623-1641 (2002).
8. Darden, T., York, D., and Pedersen, L. Particle mesh Ewald: An N · log (N) method for Ewald sums in large systems. *J. Chem. Phys.* **98**, 10089-10092 (1993).
9. Ryckaert, J.-P., Ciccotti, G., and Berendsen, H. J. Numerical integration of the cartesian equations of motion of a system with constraints: molecular dynamics of n-alkanes. *J. Comput. Phys.* **23**, 327-341 (1977).
10. Miller III, B. R., et al. MMPBSA. py: an efficient program for end-state free energy calculations. *J Chem Theory Comput.* **8**, 3314-3321 (2012).
11. Gohlke, H., and Case, D. A. Converging free energy estimates: MM-PB (GB) SA studies on the protein–protein complex Ras–Raf. *J Comput Chem.* **25**, 238-250 (2004).
12. Wang, E., et al. End-point binding free energy calculation with MM/PBSA and MM/GBSA: strategies and applications in drug design. *Chem Rev.* **119**, 9478-9508 (2019).
